# Formyl-methionyl-leucyl-phenylalanine–Induced Dopaminergic Neurotoxicity via Microglial Activation: A Mediator between Peripheral Infection and Neurodegeneration?

**DOI:** 10.1289/ehp.11031

**Published:** 2008-01-30

**Authors:** Xi Gao, Xiaoming Hu, Li Qian, Sufen Yang, Wei Zhang, Dan Zhang, Xuefei Wu, Alison Fraser, Belinda Wilson, Patrick M Flood, Michelle Block, Jau-Shyong Hong

**Affiliations:** 1 Neuropharmacology Section, Laboratory of Pharmacology and Chemistry, National Institute of Environmental Health Sciences, National Institutes of Health, Department of Health and Human Services, Research Triangle Park, North Carolina, USA; 2 Comprehensive Center for Inflammatory Disorders, University of North Carolina, Chapel Hill, North Carolina, USA

**Keywords:** fMLP, inflammation, microglia, NADPH oxidase, neurotoxicity

## Abstract

**Background:**

Parkinson disease (PD), a chronic neurodegenerative disease, has been proposed to be a multifactorial disorder resulting from a combination of environmental mechanisms (chemical, infectious, and traumatic), aging, and genetic deficits. Microglial activation is important in the pathogenesis of PD.

**Objectives:**

We investigated dopaminergic (DA) neurotoxicity and the underlying mechanisms of formyl-methionyl-leucyl-phenylalanine (fMLP), a bacteria-derived peptide, in relation to PD. METHODS: We measured DA neurotoxicity using a DA uptake assay and immunocytochemical staining (ICC) in primary mesencephalic cultures from rodents. Microglial activation was observed via ICC, flow cytometry, and superoxide measurement.

**Results:**

fMLP can cause selective DA neuronal loss at concentrations as low as 10^−13^ M. Further, fMLP (10^−13^ M) led to a significant reduction in DA uptake capacity in neuron/glia (N/G) cultures, but not in microglia-depleted cultures, indicating an indispensable role of microglia in fMLP-induced neurotoxicity. Using ICC of a specific microglial marker, OX42, we observed morphologic changes in activated microglia after fMLP treatment. Microglial activation after fMLP treatment was confirmed by flow cytometry analysis of major histocompatibility antigen class II expression on a microglia HAPI cell line. Mechanistic studies revealed that fMLP (10^−13^ M)-induced increase in the production of extracellular superoxide from microglia is critical in mediating fMLP-elicited neurotoxicity. Pharmacologic inhibition of NADPH oxidase (PHOX) with diphenylene-iodonium or apocynin abolished the DA neurotoxicity of fMLP. N/G cultures from PHOX-deficient (gp91^PHOX−/ −^) mice were also insensitive to fMLP-induced DA neurotoxicity.

**Conclusion:**

fMLP (10^−13^ M) induces DA neurotoxicity through activation of microglial PHOX and subsequent production of superoxide, suggesting a role of fMLP in the central nervous system inflammatory process.

Parkinson disease (PD), a chronic neuro-degenerative disease, is characterized by progressive and selective degeneration of dopaminergic (DA) neurons in the substantia nigra (Leenders and Oertel 2001). Although the etiology of PD is still unknown, many environmental toxins and other environmental pathomechanisms (pharmacologic, infectious, and traumatic) have been implicated. Arai et al. (2006) suggested that infection and inflammatory reaction potentially contribute to the pathogenesis of PD.

Inflammatory mediators and cellular processes once thought to be restricted to the peripheral immune system are now known to be vital in the progression of PD (Block et al. 2007; Hirsch et al. 1998; McGeer et al. 1988). Microglia, the resident immune cells and major phagocytes in the brain, have recently gained prominence as a key mediator in the inflammatory process of PD. Massive microglial activation in the substantia nigra and striatum has been observed in brains from patients with PD (McGeer et al. 1988). Activated microglia exert cytotoxic effects by releasing inflammatory mediators, such as reactive oxygen species (ROS) and proinflammatory factors (Block et al. 2007). Large amounts of these substances are toxic to adjacent neurons, and the continued production of these mediators by activated microglial cells can lead to chronic degeneration of DA neurons, as seen in progressive PD.

Activation of microglia can be regulated by both endogenous immune modulators and exogenous substances (Block et al. 2007). Previously we identified several endogenous neuropeptides, such as substance P, dynorphin, and pituitary adenylate cyclase-activating polypeptide, that can modulate microglial activity and DA neuron survival in sub-picomolar to femtomolar concentrations (Block et al. 2006; Yang et al. 2006) in rodent midbrain neuron/glia (N/G) cultures. We performed pharmacophore perception to search for other naturally occuring peptides that are chemically related to these neuropeptides to determine if they exhibit similar neuro-protective or neurotoxic functions. We found that formylmethionyl-leucyl-phenylalanine (fMLP), a bacterial-derived chemoattractant, has high chemical similarity with substance P. Several chemical features shared between fMLP and substance P, as deduced by the Catalyst/HipHop program (Accelrys, Inc., San Diego, CA), include a hydrogen bond acceptor, a hydrogen bond donor, a positively ionizable region, and a hydrophobic group (Figure 1). Based on this result, fMLP might share similar binding properties to microglia as substance P and hence trigger similar biological pathways. This discovery prompted us to speculate that fMLP might be another peptide that can affect DA neuron toxicity in a microglia-dependent manner.

fMLP, an *N*-formylated peptide, is a potent chemoattractant molecule released from both bacteria and damaged mitochondria, and it plays an important role in host defense against microbial infection or tissue injury by recruiting phagocytes to the site of inflammation. fMLP may exist in the brain in some infectious diseases of the central nervous system (CNS), such as meningitis. Circulating fMLP released from bacteria may also enter the brain through areas with a less tight blood–brain barrier, such as the circumventricular organs (Duvernoy and Risold 2007). Two functional formyl peptide receptors (FPRs), designated FPR and FPRL1R, bind fMLP with high (FPR, in the low nanomolar or picomolar range) and low (FPRL1R, in the micromolar range) affinity, respectively (Hartt et al. 1999). Expression of FPR has been observed in rodent microglia (Lorton et al. 2000). However, microglial cells lack the capacity to migrate in response to fMLP (Yao et al. 1990), suggesting that the expression of FPRs on microglia is either absent or too low to function. Therefore, in spite of all the studies on fMLP in phagocytes during the peripheral inflammatory response, little is known about the function of fMLP in the CNS inflammatory process.

In the present study, we investigated the function of fMLP in neurodegeneration, using the well-established primary rat and mouse midbrain culture model of PD. Our results showed that a subpicomolar concentration of fMLP (10^−13^ M) induces oxidative stress through activation of NADPH oxidase (also called phagocyte oxidase; PHOX) in microglia, resulting in selective DA neurotoxicity. Here, for the first time, we provide evidence for an effect of fMLP in the CNS inflammatory process and propose a new potential mediator between infectious and neurodegenerative diseases in the brain.

## Materials and Methods

### Reagents

We purchased fMLP, l-leucine methyl ester hydrobrimide (LME), 3,3′-diaminobenzidine, and urea-hydrogen peroxide tablets from Sigma Chemical Co. (St. Louis, MO). Lipopolysaccharide (LPS; strain O111:B4) was purchased from Calbiochem (San Diego, CA). Cell culture ingredients were obtained from Invitrogen (Carlsbad, CA). We purchased [^3^H]DA (30 Ci/mmol) and [^3^H]γ-aminobutyric acid (GABA; 90 Ci/mmol) from PerkinElmer Life Sciences (Boston, MA). We obtained monoclonal antibodies against the CR3 complement receptor (OX-42) from Chemicon (Temecula, CA), and polyclonal antibody against Iba-1 from Wako Chemicals USA, Inc. (Richmond, VA). Vectastain avidin-biotinylated enzyme complex, biotinylated horse anti-mouse antibody, and goat anti-rabbit secondary antibody were purchased from Vector Laboratories (Burlingame, CA).

### Animals

We obtained wild-type C57BL/6J (gp91^phox+/+^) and NADPH oxidase (PHOX)-deficient (gp91^phox−/ −^) mice from the Jackson Laboratory (Bar Harbor, ME). We bred the mice to obtain timed pregnancy with accuracy of 0.5 day. Timed-pregnant Fisher F344 rats were obtained from Charles River Laboratories (Raleigh, NC). Animals were treated humanely and with regard for alleviating suffering. Housing and breeding of animals were done in accordance with National Institutes of Health guidelines (Office of Laboratory Animal Welfare 2002). All animals were housed in a specific pathogen-free facility in conditions of a constant temperature and relative humidity. Animals had *ad libitum* access to a standard laboratory chow and were kept on a 12-hr light/dark cycle.

### Mesencephalic N/G cultures

We prepared N/G cultures from the ventral mesencephalic tissues of embryonic day 13–14 rats or day 12–13 mice as described previously (Liu et al. 2000). Briefly, dissociated cells were seeded at 5 × 10^5^/well in a 24-well plate coated with poly-d-lysine. Cells were incubated in a humidified atmosphere at 37°C, with 5% CO_2_ and 95% air, and in minimal essential medium containing 10% fetal bovine serum, 10% horse serum, 1 g/L glucose, 2 mM l-glutamine, 1 mM sodium pyruvate, 100 μM nonessential amino acids, 50 U/mL penicillin, and 50 μg/mL streptomycin. Cells were ready for treatment 7 days after initial seeding. We estimated the composition of major cell types in the culture by visually counting immuno-stained cells with antibodies against cell type–specific markers: about 40–41% neurons, 11–12% microglia, 48% astrocytes, and 1% tyrosine hydroxylase-immunoreactive (TH-IR) neurons.

### Mesencephalic microglia-depleted cultures

Mesencephalic N/G cultures were prepared as described above. Two days after the initial seeding of the cells, 1.5 mM LME (final concentration) was added to the cultures and was kept in the cultures for 72 hr to deplete microglia. Seven-day-old cultures, which contain < 0.1% microglia, were used for treatment.

### [^3^H]DA or [^3^H]GABA uptake assay

We inclubated the cultures for 20 min at 37°C with 1 μM [^3^H]DA or 5 μM [^3^H]GABA in Krebs-Ringer buffer (16 mM Na_2_HPO_4_, 119 mM NaCl, 4.7 mM KCl, 1.2 mM MgSO4, 1.3 mM EDTA, pH 7.4). We measured non-specific DA uptake in the presence of 10 μM mazindol. Nonspecific uptake was blocked for GABA with 10 μM NO–711 and 1 mM β-ala-nine. After incubation, the cells were washed with ice-cold Krebs-Ringer buffer and lysed with 1 N NaOH. We mixed cell lysates with 15 mL scintillation fluid. Radioactivity was measured with a Beckman Tri-carb 2900 TR liquid scintillation counter (Beckman Coulter, Fullerton, CA). We calculated by subtracting the nonspecific counts from that of the wells without the uptake inhibitors.

### Superoxide assay

We determined the extra-cellular superoxide production by measuring the superoxide dismutase (SOD)-inhibitable reduction of the water-soluble tetrazolium salt WST-1, using modifications of a previously described method (Gao et al. 2002). Cultures were washed twice and left in 100 μL Hanks’ balanced salt solution (HBSS). We added 50μL HBSS, LPS, or fMLP, then 50 μL WST-1 (1 mM) in HBSS, with or without SOD (600 U/mL). We read the absorbance at 450 nm immediately with a Spectra Max Plus microtiter plate spectrophotometer (Molecular Devices, Sunnyvale, CA).

### Flow cytometry

Highly aggressively proliferating immortalized (HAPI) microglial cells, a microglial-like rat cell line, were seeded in 6-well plates (9.6 cm^2^) at 5 × 10^5^ cells/well and grown in Dulbecco’s minimal essential medium with 10% fetal bovine serum to 85% confluence. Cells were stimulated with LPS (10 ng/mL) or fMLP (10^−13^ M) for 48 hr, dislodged in HBSS, pelleted (1,000 × *g* for 5 min at 4°C), and resuspended in ice-cold blocking solution (HBSS containing 1% BSA) for 20 min. We washed cells and stained them for 30 min on ice with a 1:200 dilution of phycoerythrin-conjugated anti-rat OX6 (BD Pharmingen, San Diego, CA). We ran iso-type-matched controls in parallel. Cells were then washed three times and fixed. Analysis was performed on a FACScan flow cytometer using FACSDiva software (BD Biosciences, San Jose, CA).

### Immunocytochemistry (ICC)

We stained DA neurons with the antibody against tyro-sine hydroxylase. Microglia were stained with the antibody raised against OX42 or Iba-1 antigen for rat and mouse primary cultures, respectively. In brief, 3.7% formaldehyde-fixed cultures were treated with 1% hydrogen peroxide (10 min), followed by sequential incubation with blocking solution (20 min), primary antibody (overnight, 4°C), biotinylated secondary antibody (1 hr), and Vectastain ABC reagents (1 hr). After washing, the bound complex was visualized by incubating cells with 3,3′-diamino-benzidine and urea-hydrogen peroxide tablets dissolved in water. We terminated color development by removing the reagents and washing the cultures with PBS. Images were recorded with an inverted microscope (Nikon, Tokyo, Japan) connected to a charge-coupled device camera (DAGE-MTI, Michigan City, IN) operated with MetaMorph software (Universal Imaging Corporation, Downingtown, PA). Nine representative areas per well of a 24-well plate were counted under the microscope at 100× magnification.

### Pharmacophore analysis

We used the Catalyst, version 4.9.1 software (Accelrys) for conformational and pharmacophore analysis. All molecules were built within the Catalyst modeling software, and peptides were constructed as linear chains. The maximum number of conformers generated for each molecule was 250. We removed conformers with relative energy > 20 kcal/mol, and we used the best (most rigorous and most time-consuming) conformer generation method. The following chemical features were included in the development of the pharmacophore model: hydrogen bond acceptor, hydrogen bond donor, hydrophobic group, negative charge or negatively ionizable region, positively ioniz-able region, aromatic ring, and hydrophobic aromatic region.

### Statistical analysis

The data are expressed as mean ± SD. We assessed statistical significance between two groups with an analysis of variance (ANOVA), followed by Student’s *t*-test. Statistical significance between multiple groups was performed using a one-way ANOVA. A value of *p* < 0.05 was considered statistically significant.

## Results

### MLP induces selective DA neurotoxicity

**Figure 2:**
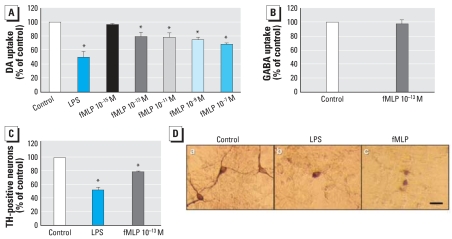
Effect of fMLP on rat primary mesencephalic N/G cultures 8 days after treatment with vehicle (control), LPS (5 ng/mL) as a positive control, or different concentrations of fMLP. (*A*) DA neurotoxicity measured using the [^3^H]DA uptake assay; values are mean ± SD from four independent experiments in triplicate. (*B*) GABA neurotoxicity measured using the [^3^H]GABA uptake assay; values are mean ± SD from three independent experiments in triplicate. (*C*) Effect of fMLP (10^−13^ M) on dopaminergic neurons observed by immunocytochemistry staining with antibody against TH; DA neurotoxicity was measured by counting TH-IR neurons 8 days after treatment. Values are mean ± SD from three independent experiments in triplicate. (*D*) Representative images shown from three separate experiments for TH-IR neurons in a control culture and in cultures treated with LPS and fMLP. Bar = 50 μm. *p < 0.05 compared with control.

We used the well-established primary mesencephalic N/G culture system as an *in vitro* model to investigate the possible role of fMLP in CNS neurodegenerative diseases. Cultures were treated with fMLP (10^−7^, 10^−9^, 10^−11^, 10^−13^, or 10^−15^ M) for 8 days. The degeneration of DA neurons was determined by [^3^H]DA uptake assay and cell count. As shown in Figure 2A, fMLP at 10^−7^ to 10^−13^ M significantly reduced the capacity of cultures to take up DA versus vehicle control. LPS, which is well known as an endotoxin and inflammatory agent, was used as a positive control to induce selective damage to DA neurons through activation of microglia. Although the regulation of many important biological functions of neutrophils requires a relatively high concentration (> 10^−7^ M) of fMLP (Balazovich et al. 1996; Elbim et al. 1993), it is rare for microglia to be exposed to such high concentrations due to the diffusion in the peripheral blood system and the difficulty of passing the blood–brain barrier. Therefore, although DA neurotoxicity was observed at higher concentrations of fMLP treatment, we focused on the effect of fMLP at 10^−13^ M, which might be close to the physically relevant concentration of fMLP in CNS. We used [^3^H]GABA uptake as an index for the selectivity of fMLP-induced toxicity. Figure 2B indicates that fMLP (10^−13^ M) failed to show any effect on [^3^H]GABA uptake capacity, indicating that fMLP is selectively toxic to DA neurons. Using ICC staining of TH, we observed morphologic changes in DA neurons after fMLP treatment (Figure 2D). The number of TH-IR neurons was counted as another index of neurotoxicity (Figure 2C). Consistent with our uptake results, fMLP (10^−13^ M) significantly reduced the number of TH-IR neurons.

### Microglia are indispensable to sub-picomolar fMLP-induced DA neurotoxicity

**Figure 3:**
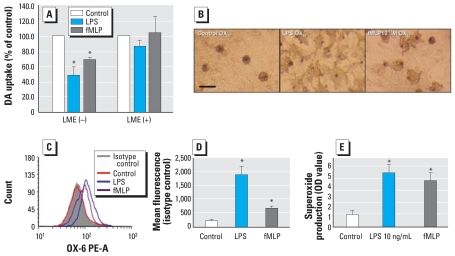
Effects of subpicomolar fMLP on microglial activation in primary rat N/G cultures (LME–) or microglia-depleted cultures (LME+) treated with vehicle, LPS (5 ng/mL; positive control for microglia activation), or fMLP (10^−13^ M). (*A*) DA neurotoxicity measured using the [^3^H]DA uptake assay 8 days after treatment; values are mean ± SD from three independent experiments in triplicate. (*B*) Activation of microglia visualized by immunocytochemical staining using OX-42 antibody 24 hr after LPS (5 ng/mL) or fMLP (10^−13^ M) treatment in rat mecencephalic N/G cultures. Representative images are shown from three separate experiments; bar = 50 μm. (*C, D*) Expression of MHCII (OX-6) in the HAPI rat microglial line treated with LPS (10 ng/mL) or fMLP (10^−13^ M) measured by flow cytometry (*C*) and by fluorescence compared with isotype-matched controls (*D*); values are mean ± SD from three independent experiments. (*E*) Production of extra-cellular superoxide in N/G cultures from rats treated with vehicle, LPS (10 ng/mL), or fMLP (10^−13^ M) and measured by the SOD-inhibitable reduction of WST-1; values are mean ± SD from five independent experiments in triplicate. *p < 0.05 compared with control cultures.

To determine the role of microglia in fMLP-induced neurotoxicity, we treated rat primary mesencephalic N/G cultures with LME (1.5 mM) to deplete microglia. Seven days after initial seeding, cultures were treated with fMLP (10^−13^ M). Eight days after the treatment, fMLP (10^−13^ M) resulted in significant reduction of DA uptake in N/G cultures, but it failed to reduce DA uptake in microglia-depleted cultures (Figure 3A).

Morphologic changes in microglia were visualized 24 hr after treatment by ICC staining of OX-42, a specific microglial marker recognizing the CR3 receptor. We used LPS-treated cultures as a positive control to show the morphologic change of microglial activation. As shown in Figure 3B, N/G cultures treated with fMLP (10^−13^ M) displayed intensified OX-42 immunoreactivity compared with vehicle control. OX-42-positive microglia cells demonstrated characteristics of activation, including increased cell size and amoeboid-like shape.

We also used HAPI rat microglia cells to determine the effect of 10^−13^ M fMLP on the expression of immune markers of microglia. We measured expression of major histo-compatibility antigen class II (MHCII, OX-6) by flow cytometry analysis 24 hr after treatment (Figure 3C). MHC-II was chosen as a marker for microglia activation because MHCII-positive activated microglia in the substantia nigra may be involved in progressive neurodegeneration (Yasuda et al. 2007). MHCII expression on untreated HAPI cells is very low; fMLP (10^−13^ M) treatment significantly increased the expression of MHCII compared with control cultures. Taken together, the preceding data demonstrate that microglia were activated after 10^−13^ M fMLP treatment and that activated microglia play an indispensable role in subpicomolar fMLP-induced DA neurotoxicity.

### Subpicomolar fMLP induces production of superoxide from microglia

Oxidative stress has been frequently observed in several neurologic disorders, including PD (Jenner 2003). Micromolar concentrations of fMLP have been shown to stimulate the production of superoxide anion in neutrophils (Dewald and Baggiolini 1985; Sakamoto et al. 2006). In the present study, we investigated whether fMLP can induce the production of super-oxide in microglia. N/G cultures were seeded in 96-well plates and then treated with vehicle, LPS (10 ng/mL), or fMLP (10^−13^ M). The generation of extracellular superoxide was measured as SOD-inhibitable reduction of WST-1. fMLP (10^−13^ M) significantly stimulated the release of extracellular superoxide (Figure 3D) compared with vehicle control.

### Microglial PHOX is a key contributor mediating neurotoxicity of subpicomolar fMLP

**Figure 4:**
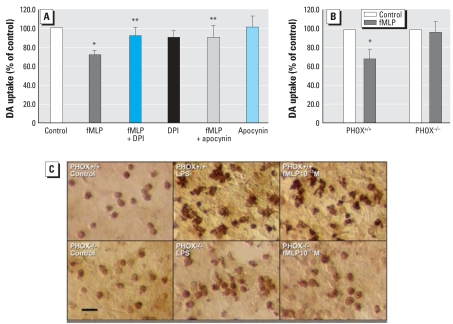
Role of microglial PHOX in subpicomolar fMLP-induced DA neurotoxicity in mesencephalic rat primary N/G cultures. (*A*) DA neurotoxicity determined by the [^3^H]DA uptake assay in mesencephalic N/G cultures 8 days after pretreatment with DPI (10^−9^ M) or apocynin (0.2 mM) for 30 min and treatment with fMLP (10^−13^ M); values are mean ± SD of at least three separate experiments in triplicate. (*B*) DA neurotoxicity determined by the [^3^H]DA uptake assay in mesencephalic N/G cultures from gp91^PHOX+/+^ and gp91^PHOX−/ −^ mice treated with vehicle or fMLP (10^−13^ M) for 8 days; values are mean ± SD from three independent experiments in triplicate. (*C*) Activation of microglia visualized by immunostaining of Iba-1 antigen in mesencephalic N/G cultures from gp91^PHOX+/+^ and gp91^PHOX−/ −^ mice treated with vehicle, LPS (5 ng/mL), or fMLP (10^−13^ M) for 24 hr. Images are representative of three independent experiments; bar = 50 μm. *p < 0.05 compared with control cultures. **p < 0.05 compared with fMLP-alone-treated cultures.

NADPH oxidase (PHOX) is the major enzyme involved in the production of extracellular superoxide in phagocytes (Babior 1999). To determine whether microglial PHOX plays an important role in fMLP-induced neurotoxicity, we pharmacologically inhibited microglial PHOX in primary rat mesencephalic N/G cultures using diphenylene-iodonium (DPI) or apocynin. We used 10^−9^ M DPI because DPI at this concentration is effective at inhibiting PHOX activity without any observable neuro-toxicity (Qin et al. 2002). Cultures were pre-treated with or without DPI (10^−9^ M) or apocynin (0.2 mM) for 30 min before fMLP (10^−13^ M) treatment. We determined DA neurodegeneration 8 days after treatment by [^3^H]DA uptake assay. Both DPI and apocynin significantly attenuated fMLP (10^−13^ M)-induced neurotoxicity, with 21% and 19% increases in DA uptake capacity, respectively, compared with fMLP (10^−13^ M) alone (Figure 4A). DPI or apocynin alone had no effect on DA uptake capacity.

To further study the role of PHOX in mediating fMLP-induced neurotoxicity, we used mutant mice deficient in gp91, the catalytic subunit of this superoxide-producing enzyme. N/G cultures from wild-type (gp91^PHOX+/+^) mice and mutant (gp91^PHOX−/ −^) mice were treated with vehicle or fMLP (10^−13^ M). Eight days later, we assessed DA neurotoxicity by [^3^H]DA uptake assay (Figure 4B). fMLP markedly decreased DA uptake capacity by 32% compared with vehicle in wild-type (gp91^PHOX+/+^) mice cultures, but it failed to show any DA neurotoxicity in mutant (gp91^PHOX−/ −^) mice cultures. Morphologic analysis of microglial activation was performed by ICC staining of Iba-1 (Figure 4C), a calcium-binding protein specific to microglia. As early as 24 hr after fMLP (10^−13^ M) treatment, activated microglia with irregular shape, enlarged cell size, and enhanced Iba-1 immunoactivity were observed in wild-type mice cultures but not in mutant mice cultures. These data confirm the critical role of microglial PHOX in DA neurotoxicity induced by subpicomolar fMLP.

## Discussion

Using primary rat mesencephalic N/G and microglia-depleted cultures, we showed that fMLP causes selective DA neurodegeneration at concentrations of 10^−7^ M to 10^−13^ M and that microglia are actively involved in the fMLP (10^−13^M)-induced DA neuronal loss. An increase in the release of superoxide anion generated by microglial PHOX plays a vital role in the neurotoxicity of subpicomolar concentrations of fMLP, as demonstrated by the reduction in fMLP-induced DA toxicity following pharmacologic inhibition or in genetically engineered knockout mice devoid of PHOX activity.

Traditionally, fMLP is recognized as a strong chemoattractant and activator of phagocytic cells in peripheral blood, leading to release of oxygen-derived free radicals, which results in removal of invading micro-organisms, thereby playing a key role in the immune response to infection and inflammation (Panaro and Mitolo 1999). fMLP can induce cell adherence and degranulation in peripheral phagocytic cells, such as neutrophils, in the blood (Videm and Strand 2004). fMLP triggers the FPRs on neutrophils to activate PHOX, generating bactericidal super-oxide anion and proinflammatory cytokines (Dewald and Baggiolini 1985; Sakamoto et al. 2006). However, studies on the effect of fMLP on microglia have been limited due to the fact that microglial cells lack the capacity to migrate in response to fMLP (Yao et al. 1990). Interestingly, our pharmacophore analysis illustrates that fMLP and substance P share common chemical features, and previous studies by our group indicated that sub-picomolar concentrations of substance P are selectively toxic to DA neurons through microglia-generated oxidative stress responses (Block et al. 2006). Thus, we hypothesized that fMLP, although lacking activity as a chemoattractant for microglia, may activate microglia and cause inflammation-related DA toxicity similarly to substance P. The results given here demonstrate that fMLP (10^−13^ M) is selectively toxic to DA neurons after 8 days of treatment in primary rat mesencephalic N/G cultures (Figure 2). We conclude that the neurotoxicity observed in these cultures is mediated by microglial cells based on the following observations: *a*) Microglia were found to be activated 24 hr after fMLP (10^−13^ M) treatment, changing from resting ramified microglia into activated amoeboid ones (Figure 3B); *b*) the membrane expression of MHCII (OX-6), a marker of activated microglia, increased significantly after fMLP (10^−13^ M) treatment (Figure 3C); and *c*) fMLP (10^−13^ M) failed to induce degeneration of DA neurons in microglia-depleted cultures (Figure 3A), suggesting an indispensable role of microglia in subpicomolar fMLP-induced DA neurodegeneration.

Activated microglia can produce and release a large number of proinflammatory factors, including free radicals such as superoxide (Roepstorff et al. 2007) and nitric oxide (Liu et al. 2002), as well as proinflammatory cytokines, such as interleukin (IL)-1β and tumor necrosis factor (TNF)-α (Mogi et al. 1996; Nagatsu and Sawada 2007). Although these immunotoxic factors are necessary for normal function, the microglial response must be tightly regulated to avoid overactivation and disastrous neurotoxic consequences. Overproduction of ROS from microglia may be toxic to DA neurons through two major pathways: *a*) Production of extracellular superoxide is directly toxic to DA neurons; and *b*) the generated superoxide can subsequently be dismutated to cell-permeable hydrogen peroxide (Paravicini et al. 2004), resulting in an increase in intracellular ROS. Intracellular ROS in microglia is a critical component of pro-inflammatory signaling and amplifies the production of neurotoxic proinflammatory factors, such as nitric oxide and TNF-α (Block et al. 2007). In the present study, 10^−13^ M fMLP significantly stimulated microglia to release extra-cellular superoxide compared with vehicle (Figure 3D). The microglial production of extracellular superoxide might be responsible for the subpicomolar fMLP-induced neurotoxicity. Intracellular ROS levels were also measured in enriched microglia cultures after stimulation with fMLP (10^−13^ M), showing a slight increase in fluorescent signal compared with the vehicle cultures (data not shown). Therefore, production of intracellular ROS and its downstream reactions may also be involved in fMLP-induced neurotoxicity, but to a lesser extent. The production of nitric oxide and TNF-α were below the detection limits of the assays after fMLP (10^−13^ M) treatment (data not shown). However, we cannot rule out the possibility of their contribution to fMLP-induced DA neurotoxicity. Low quantities of nitric oxide, for instance, may react with super-oxide free radicals to form peroxynitrite, which is highly neurotoxic (Beckman et al. 1993). The exact mechanisms by which fMLP exerts its neurotoxic effect on DA neurons through microglial activation remains to be determined.

Data from this study suggest that PHOX is critical for subpicomolar fMLP regulation of microglial activation and vital in fMLP-induced DA neurotoxicity. Pharmacologic inhibition of PHOX or genetic knockout of the gp91 subunit of PHOX completely abolished fMLP-induced DA neurotoxicity (Figure 4). However, whether fMLP (10^−13^ M) mediates glial cell activation by directly interacting with PHOX or indirectly through the FPRs is not clear. There is evidence that fMLP may activate the p38 mitogen-activated protein kinase or phosphatidylinositol 3-kinase signal transduction pathways via the activation of FPRs and lead to the activation of neutrophil PHOX (Fu et al. 2004). Accumulating evidence indicates that the expression of high-affinity FPR in microglial cells is subject to up-regulation by both pathogen- and host-derived molecules associated with inflammatory responses such as LPS (Cui et al. 2002a), and the proinflammatory cytokines TNF-α (Cui et al. 2002b) and interferon-γ (Chen et al. 2007). However, high-affinity FPR binds and is activated by fMLP in picomole to low nano-mole concentrations (Le et al. 2000). There are no reports of FPRs being activated by sub-picomolar concentrations of fMLP. In fact, many femtomolar-acting compounds tested in our laboratory, such as substance P, dynorphin (Block et al. 2006), naloxone (Qin et al. 2005), and dextromethorphan (Liu et al. 2001) are independent of traditional receptor-mediated mechanisms. PHOX seems to be a common pathway for these femtomolar-acting compounds. Ongoing research in our laboratory is trying to elucidate the action site of sub-picomolar fMLP. Given the importance of PHOX in the effect of all the femtomolar-acting compounds we tested, it seems likely that PHOX is a potential target for fMLP.

PD has been proposed to be a multi-factorial disease resulting from a combination of normal aging, genetic deficits, and environmental injuries (Snyder and Adler 2007). The impact of systemic infection on the progression of neurodegenerative diseases has been documented (Arai et al. 2006; Combrinck et al. 2002; Panitch 1994), and several circulating cytokines (interferon-γ and TNF-α) (Perry et al. 2003; Qin et al. 2007) have been proposed to be important mediators. In the present study, we demonstrated that sub-picomolar fMLP induces specific degeneration to DA neurons and thus might serve as a mediator between infectious diseases and neurodegeneration. fMLP may constantly gain entrance into the CNS through circumventricular organs (Duvernoy et al. 2007) during chronic peripheral infectious diseases when there is persistent circulating fMLP. Although the infiltrated fMLP in this condition might not be as high as it is in the blood, or even under detectable levels, it could still be a potential threat to DA neurons because fMLP is toxic to DA neurons at concentration as low as 10^−13^ M. In addition, in a number of acute cerebral pathologies, including stroke, trauma, and meningitis, the blood–brain barrier permeability increases, allowing plasma proteins to enter the brain parenchyma. In PD patients, dysfunction of the blood–brain barrier was also observed (Kortekaas et al. 2005). Because the peripheral circulating fMLP is a small molecular-weight tripeptide, it is likely able to pass through the blood–brain barrier during inflammation. fMLP-induced PMN activation resulted in an increase in endothelial cell permeability (Gautam et al. 1998). This may also occur to the blood–brain barrier during the process of inflammation, which would further facilitate fMLP penetration. Given all these possibilities for fMLP to enter the CNS and the innate vulnerabilities of the nigrostriatal dopaminergic system (Block et al. 2007), the results of the present study highlight the potential role of infectious agents in neurodegenerative diseases and may aid in the development of new therapies.

## Figures and Tables

**Figure 1:**
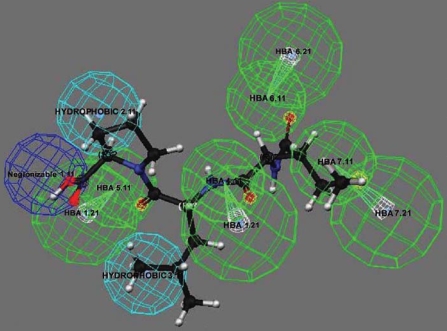
Pharmacophore analysis between fMLP and substance P. Similar chemical features shared by these two peptides are illustrated by three-dimensional relationships with the highest fit values. fMLP resembles the amino acid sequence (Phe-Phe-Gly-Leu-Met) of the C-terminus of substance P. Hydrogen bond acceptor (HBA), green; negative ionizable, dark blue; hydrophobic, light blue.
